# Melatonin as a Guardian of Mitochondria: Mechanisms and Therapeutic Potential in Neurodegenerative Diseases

**DOI:** 10.3390/biology15020189

**Published:** 2026-01-20

**Authors:** Yanyu Bao, Guoying Miao, Nannan He, Xingting Bao, Zheng Shi, Cuilan Hu, Xiongxiong Liu, Bing Wang, Chao Sun

**Affiliations:** 1 Institute of Modern Physics, Chinese Academy of Sciences, Lanzhou 730000, China; baoyanyu@impcas.ac.cn (Y.B.); hucuilan@impcas.ac.cn (C.H.); lxx002@impcas.ac.cn (X.L.); 2 State Key Laboratory of Heavy Ion Science and Technology, Institute of Modern Physics, Chinese Academy of Sciences, Lanzhou 730000, China; 3 University of Chinese Academy of Sciences, Beijing 100049, China; 4 Gansu Provincial Maternity and Child-Care Hospital/Gansu Provincial Central Hospital, Lanzhou 730000, China; miaoguoying@gszy.edu.cn; 5 Department of Critical Care Medicine, The Second Hospital of Lanzhou University, Lanzhou 730000, China; ldyy_henn@lzu.edu.cn; 6 Medical College, Northwest Minzu University, Lanzhou 730000, China; baoxingting@xbmu.edu.cn; 7 School of Biopharmaceutical and Engineering, Lanzhou Jiaotong University, Lanzhou 730070, China; shizheng@lzjtu.edu.cn; 8 Institute for Radiological Science, National Institutes for Quantum Science and Technology (QST), Chiba 263-8555, Japan

**Keywords:** neurodegenerative diseases, mitochondrial dysfunction, melatonin, mitochondrial quality control, oxidative stress

## Abstract

Mitochondrial dysfunction is a key early pathological process in neurodegenerative diseases (NDs), leading to oxidative stress, impaired energy metabolism, and neuronal apoptosis prior to the onset of clinical symptoms. Although mitochondria represent important therapeutic targets, effective interventions targeting mitochondrial function remain limited. This review summarizes current evidence regarding the mechanisms by which melatonin protects mitochondria and evaluates its therapeutic relevance, with a primary focus on Alzheimer’s disease, Parkinson’s disease, and Huntington’s disease—the major protagonists of NDs—while briefly covering other NDs such as amyotrophic lateral sclerosis, multiple sclerosis, and prion diseases. Melatonin selectively accumulates in neuronal mitochondria and exerts neuroprotection through multiple pathways: (1) direct scavenging of reactive oxygen species (ROS); (2) transcriptional activation of antioxidant defenses via the SIRT3 and Nrf2 pathways; (3) regulation of mitochondrial dynamics through DRP1 and OPA1; and (4) promotion of PINK1- and Parkin-mediated mitophagy. Additionally, melatonin exhibits context-dependent pleiotropy: under conditions of mild mitochondrial stress, it restores mitochondrial homeostasis; under conditions of severe mitochondrial damage, it promotes pro-survival autophagy by inhibiting the PI3K/AKT/mTOR pathway, thereby conferring stage-specific therapeutic advantages. Overall, melatonin offers a sophisticated mitochondria-targeting strategy for the treatment of NDs. However, successful clinical translation requires clarification of receptor-dependent signaling pathways, development of standardized dosing strategies, and validation in large-scale randomized controlled trials.

## Simple Summary

Neurodegenerative diseases such as Alzheimer’s and Parkinson’s are becoming increasingly important global health concerns as population ageing accelerates. A central early event in these disorders is mitochondrial dysfunction, where impairment of the cell’s “powerhouse” initiates a harmful cycle of oxidative stress and progressive neuronal loss. Beyond its well-recognized role in sleep regulation, melatonin has shown strong potential for protecting mitochondria. It can readily enter the brain, accumulate within mitochondria, neutralize reactive oxygen species, strengthen endogenous antioxidant systems, preserve mitochondrial structure, and remove irreversibly damaged mitochondria through autophagy. Although clinical application is still limited by uncertainties regarding optimal dosage and the lack of large-scale studies, melatonin represents a promising multi-target therapeutic approach with the potential to slow the progression of neurodegenerative diseases.

## 1. Introduction

Neurodegenerative diseases (NDs), such as Alzheimer’s disease (AD), Parkinson’s disease (PD), and Huntington’s disease (HD), are chronic and progressive disorders characterized by gradual loss of neuronal structure and function. With rapid global population ageing, the prevalence of NDs has increased markedly, making them a major health burden for older adults. Mitochondria, which function as the cellular “powerhouses” as well as signaling hubs, play essential roles in energy production, redox balance, and regulation of cell survival and death. Although individual NDs are associated with distinct pathogenic proteins, such as β-amyloid in AD and α-synuclein (α-syn) in PD, accumulating evidence indicates that mitochondrial dysfunction represents a shared and early pathological process that arises long before clinical symptoms appear [1,2]. Impairment of the mitochondrial respiratory chain results in excessive production of ROS, reduced ATP generation, abnormalities in mitochondrial dynamics, and disruption of mitochondrial quality control system. These alterations promote oxidative stress, synaptic dysfunction, and neuronal apoptosis, establishing a self-amplifying pathogenic cascade [3].

Despite the central role of mitochondrial dysfunction in NDs, therapeutic strategies that effectively target mitochondrial impairment remain limited. Melatonin, an endogenous indoleamine best known for its regulation of circadian rhythm, has gained increasing attention for its strong mitochondrion-protective properties. Melatonin readily crosses cellular membranes and accumulates within multiple subcellular compartments, including mitochondria [4]. Its neuroprotective actions involve several complementary mechanisms. Melatonin directly scavenges mitochondrial ROS and reduces oxidative injury to mitochondrial DNA (mtDNA), lipids, and proteins [5]. It enhances endogenous antioxidant defense through activation of SIRT3 and Nrf2 signaling pathways [6,7]. In addition, melatonin regulates mitochondrial dynamics by modulating the activity of DRP1 and OPA1, thereby preserving mitochondrial network integrity [8]. Furthermore, melatonin promotes PINK1 and Parkin-mediated autophagy, facilitating selective removal of damaged mitochondria and preventing the release of pro-apoptotic factors [9].

A distinctive feature of melatonin is its context-dependent bidirectional regulation of mitochondrial protection. Under physiological conditions or mild mitochondrial stress, melatonin primarily restores mitochondrial function by eliminating excess ROS and reinforcing antioxidant systems [10]. Under severe mitochondrial damage, melatonin shifts its predominant action toward activation of protective autophagy through inhibition of the PI3K/AKT/mTOR pathway, thereby promoting the selective removal of irreversibly impaired mitochondria [11]. This dual regulatory capacity highlights its potential to provide therapeutic benefit across different stages of ND progression.

Although preclinical studies demonstrate strong therapeutic promise, the clinical translation of melatonin for NDs faces several challenges. The relative contributions of receptor-dependent and receptor-independent pathways remain to be clarified. Standardized dosing strategies and optimal treatment timing have not yet been established, and evidence from large-scale randomized controlled trials is still limited. Therefore, a comprehensive understanding of the molecular pathways through which melatonin regulates mitochondrial function is critical for advancing precise mitochondrion-targeted therapeutic strategies. This review aims to elucidate the relationship between melatonin and mitochondrial function, summarize its core protective mechanisms in AD, PD, and HD, and discuss the clinical translation and future development of melatonin-based therapies.

## 2. Melatonin Uptake and Synthesis

Melatonin is an amine hormone primarily produced by the pineal gland and is widely distributed across living organisms. Known as the “hormone of darkness” because of its central role in regulating circadian rhythms and sleep–wake cycles, melatonin is chemically identified as N-acetyl-5-methoxytryptamine and is also referred to as the pineal hormone. Its secretion follows a distinct circadian rhythm and influences a broad range of physiological processes. Although the pineal gland is the major source of circulating melatonin, peripheral tissues and cells, including the skin, gastrointestinal tract, thymus, bone marrow, and lymphocytes, also produce small amounts. Due to its strong lipophilicity, melatonin readily crosses the blood–brain barrier, enters systemic circulation, and exerts regulatory effects across multiple organ systems [12].

### 2.1. Pineal Melatonin Biosynthesis

**Figure 1:**
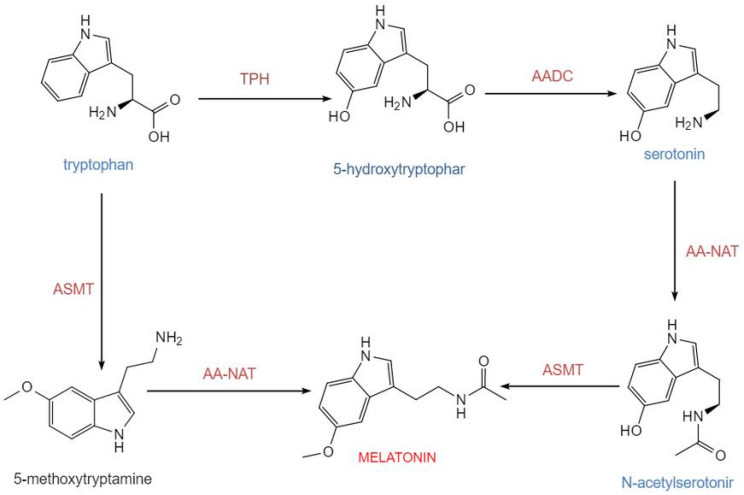
Melatonin biosynthesis occurs via two distinct pathways. Serotonin can first be catalyzed by AA-NAT to form N-acetylserotonin, which is subsequently converted to melatonin by ASMT. Alternatively, serotonin can first be methylated by ASMT to produce 5-methoxytryptamine, which is then acetylated by AA-NAT to yield melatonin. Together, these two pathways constitute a branched network for melatonin synthesis. Abbreviations: TPH, tryptophan-5-hydroxylase; AADC, L-aromatic amino acid decarboxylase; AA-NAT, arylalkylamine N-acetyltransferase; ASMT, N-acetylserotonin-O-methyltransferase. This figure was created with InDraw.

The biosynthesis of melatonin in pinealocytes originates from the essential amino acid tryptophan. In mammals, including humans, this synthetic pathway consists of four consecutive major enzymatic steps: tryptophan is first hydroxylated by tryptophan-5-hydroxylase (TPH) to generate 5-hydroxytryptophan, which is subsequently decarboxylated by aromatic-L-amino acid decarboxylase (AADC) into 5-hydroxytryptamine (serotonin). Serotonin is then acetylated by arylalkylamine N-acetyltransferase (AA-NAT) to form N-acetylserotonin, which is finally methylated by acetylserotonin O-methyltransferase (ASMT) to produce melatonin. This route constitutes the core process of melatonin generation in mammals [13] (Figure 1). Newly synthesized melatonin is rapidly released into the circulation and distributed to central and peripheral tissues. Its synthesis is tightly regulated by the light-dark cycle, with darkness promoting and light inhibiting production [14]. Tryptophan-5-hydroxylase and aromatic-L-amino acid decarboxylase determine the basal supply of serotonin, while AA-NAT and ASMT are the key enzymatic systems governing the final output of melatonin. Experimental data indicate that in the rat pineal gland, the diurnal rhythm of AA-NAT activity exhibits an approximately 150-fold variation, underscoring its role as the primary rate-limiting enzyme in circadian synthesis. Although the rhythmic change in ASMT activity is relatively modest, it remains indispensable for the overall synthetic process [15].

This biosynthetic process is stringently controlled by the light-dark cycle via neuroendocrine mechanisms. Photonic information perceived by the retina is relayed via the retinohypothalamic tract to the suprachiasmatic nucleus (SCN) of the hypothalamus. The SCN regulates the pineal gland through sympathetic output, resulting in the circadian release of norepinephrine (NE). Norepinephrine, with β_1_-adrenergic receptor activation serving as an essential foundation and α_1_-adrenergic receptors synergistically enhancing the β_1_-mediated elevation of intracellular cAMP signaling, significantly increases AA-NAT activity, thereby accelerating melatonin synthesis [16,17]. Correspondingly, the diurnal rhythm of melatonin synthesis is particularly pronounced: during the night (the dark phase of the light-dark cycle), AA-NAT activity in the pineal gland rises markedly, leading to increased melatonin synthesis and its subsequent release into the bloodstream. In contrast, during the daytime (the light phase), synthesis is suppressed, maintaining low plasma melatonin concentrations. The nocturnal serum peak of melatonin typically occurs during the late night (e.g., 2:00–4:00 a.m.), while daytime levels remain low [18,19]. Further mechanistic studies have revealed that AA-NAT activity is closely associated with its binding to 14-3-3 proteins, cAMP-regulated signaling pathways, and protein degradation mechanisms. These post-transcriptional and post-translational regulatory processes are critical for circadian synthesis [20].

Within the broader context of biological evolution, the melatonin synthesis pathway is conserved across diverse kingdoms of life. For instance, plants and microorganisms are also capable of generating melatonin from tryptophan. Although the specific enzyme systems may differ, they are functionally homologous, reflecting the widespread preservation of melatonin’s fundamental roles as a signaling molecule and antioxidant in living organisms [21].

### 2.2. Mitochondrial Targeting and Uptake of Melatonin

Melatonin is a highly conserved antioxidant with strong mitochondrial targeting properties. This targeting arises from both active transport into mitochondria and potential local synthesis within these organelles [22,23,24,25,26]. These mechanisms ensure high intramitochondrial concentrations, allowing melatonin to effectively mitigate oxidative stress and preserve mitochondrial integrity. Because mitochondria are major sites of ROS generation, their localized enrichment of melatonin is crucial for preventing oxidative damage. The presence of melatonin within mitochondria allows for the direct neutralization of free radicals, thereby preventing cellular injury induced by ROS [27]. Building on this concept, recent investigations have begun to define the specific pathways through which melatonin accumulates within mitochondria, offering new insight into its roles in neuroprotection, aging, and metabolic diseases (Table 1).

The entry of melatonin into mitochondria is not merely passive diffusion but relies on specific transport proteins that facilitate its efficient accumulation. Although traditionally viewed as freely membrane-permeable due to its lipophilicity, emerging evidence indicates that melatonin, as an amphiphilic molecule, can passively diffuse into cells and tissues [28]. However, an emerging concept in formation suggests that the entry of melatonin into the mitochondrial matrix to scavenge hydrophilic ROS depends on active transport, with key transporters including the oligopeptide transporters PEPT1 and PEPT2 (SLC15A1/2). Recent studies have further confirmed through cancer cell immunofluorescence co-localization experiments that PEPT1/2 are present on the mitochondrial membrane and directly participate in the mitochondrial transport process of melatonin [22]. In isolated mouse brain and liver mitochondria, supplementation with 100 nM melatonin led to intramitochondrial concentrations up to 100-fold higher than plasma levels, attributable to oligopeptide transporter (PEPT1/2)-mediated uptake and/or local mitochondrial synthesis [23,24]. Indeed, mitochondria harbor key melatonin-synthesizing enzymes AA-NAT and ASMT [25,26]. Notably, pinealectomy almost completely abolishes blood melatonin, yet tissue melatonin levels remain unaffected, suggesting that peripheral tissue (including mitochondrial) melatonin is maintained independently of pineal sources, likely through compensatory uptake or local synthesis [29]. Furthermore, mitochondrial melatonin levels exhibit saturation kinetics, indicating finite transport capacity [28]. These findings underscore that mitochondrial enrichment of melatonin represents an evolutionarily conserved strategy underpinning its neuroprotective functions [30].

### 2.3. Evidence for Intramitochondrial Melatonin Synthesis

As the central site of cellular energy metabolism, mitochondria not only generate ROS but also serve as a key site for endogenous melatonin synthesis. In addition to exogenous cellular uptake, growing experimental evidence indicates that mitochondria possess the capability for endogenous melatonin synthesis. Mitochondrially derived melatonin constitutes an important complement independent of pineal sources. This conclusion is grounded in evolutionary biology and the endosymbiotic theory: melatonin is an ancient molecule whose origins trace back to early life forms; its presence and synthetic activity have been detected in α-proteobacteria such as purple non-sulfur bacteria (*Rhodospirillum rubrum*) and in photosynthetic cyanobacteria [31]. According to the endosymbiotic hypothesis, ancestral eukaryotes engulfed such bacteria, with purple non-sulfur bacteria evolving into mitochondria and cyanobacteria into chloroplasts; these organelles retained their ancestral melatonin-synthetic capacity. In plants, which possess both mitochondria and chloroplasts as dual synthesis sites, melatonin levels are typically significantly higher than in animal cells containing only mitochondria, further supporting the primordial role of organelle-derived melatonin [32]. Numerous *in vitro* studies have confirmed the capacity of mitochondria to synthesize melatonin. Experiments using isolated mouse oocyte mitochondria directly demonstrated the production of melatonin upon supplementation with serotonin, and incubation studies with brain mitochondria further verified that these organelles can synthesize melatonin via endogenous biochemical pathways [26,33]. *In vitro* maturation experiments of porcine oocytes, employing immunofluorescence staining and confocal microscopy, confirmed the expression of the key melatonin-synthesizing enzymes AA-NAT and ASMT in porcine oocytes, with their localization overlapping with mitochondrial markers. Immunoelectron microscopy further revealed that AA-NAT is predominantly distributed within mitochondria, with a portion also present in the cytoplasm. In culture systems supplemented with serotonin, ultra-high-performance liquid chromatography-tandem mass spectrometry (UPLC-MS/MS) analysis detected significantly higher melatonin levels in the supernatant of mitochondrial cultures compared to controls. Furthermore, this synthetic output increased progressively during oocyte maturation from the MI to MII stage [34]. These findings indicate that the mitochondria of porcine oocytes not only contain the key enzymes required for melatonin synthesis but are also capable of autonomously producing melatonin when provided with appropriate precursors. Moreover, this synthesis process is closely correlated with the progression of oocyte maturation. Additionally, kinetic analyses revealed that mitochondria, due to their abundant substrate supply, convert serotonin to melatonin with higher efficiency [35]. The intramitochondrial concentration of melatonin is significantly higher than in other subcellular compartments and does not fluctuate with serum melatonin levels, further substantiating the autonomy and independence of its local synthesis [28]. In brain tissue, melatonin is synthesized specifically at the mitochondrial level and functions locally without being released into the systemic circulation. The phenomenon where circulating melatonin levels decline after pinealectomy while intramitochondrial melatonin levels increase also suggests the existence of compensatory local synthesis [26]. These discoveries indicate that mitochondrial synthesis of melatonin may represent an autonomous, rapid-response mechanism against oxidative stress, rather than being dependent on external supply.

While evidence confirms that mitochondria can produce melatonin, this does not preclude the possibility of cytoplasmic synthesis—erythrocytes, which lack mitochondria, are also capable of producing melatonin [36]. N-acetyl-CoA, an indispensable substrate for AA-NAT, is primarily synthesized within mitochondria. From the perspective of substrate availability, the efficiency of melatonin synthesis in the cytoplasm is significantly lower than in mitochondria. Therefore, it is proposed that under most circumstances, the amount of melatonin synthesized in the cytoplasm is negligible compared to that produced in mitochondria. Suofu et al., through protein degradation experiments, inferred that AA-NAT is localized to the mitochondrial matrix and claimed that melatonin is synthesized exclusively within the matrix [26]. Conversely, Yang et al. observed AA-NAT presence in the mitochondrial intermembrane space near the outer membrane [37]. Consequently, at present, it is challenging to definitively confine melatonin synthesis strictly to either the mitochondrial matrix or the intermembrane space.

Existing evidence indicates significant sex differences in the synthesis, metabolism, and biological effects of melatonin. These differences are regulated by sex hormones such as estrogen and testosterone and are closely associated with mitochondrial function: Under normal or aging conditions, plasma melatonin and cortisol levels are significantly higher in females than in males. Even in an elderly population with an average age of 80.9 years, women maintain higher plasma melatonin levels, suggesting a greater reserve for melatonin synthesis and a stronger capacity for melatonin production under conditions of aging or stress [38,39]. In pathological states, chronic kidney disease (CKD) leads to a significant decline in plasma melatonin levels in rats, with the most pronounced decrease observed in males, followed by ovariectomized (OVX) females, and the least decline in intact females. The latter group also exhibited advantages including less reduction in renal antioxidant capacity (TAC) and smaller increases in oxidative stress markers (MDA) and inflammatory factors (TGF-β). This disparity is associated with the protective effects of female sex hormones, while male hormones may exacerbate the damage to the melatonin system caused by CKD [40]. In response to environmental light exposure, women exhibit more significant suppression of melatonin by moderate-intensity light but a lower alertness response. Their dim light melatonin onset (DLMO) occurs on average approximately 28 min earlier than in males, and DLMO in the luteal phase is earlier than in the follicular phase. This difference shows no significant association with sex hormone levels across the menstrual cycle and may stem from genetic variations or differences in photoreceptor cells [41]. In metabolic-related models, among diet-induced obese F344 rats, melatonin levels were consistently higher in females under different photoperiods with differences more pronounced under short photoperiods. The impact of photoperiod on melatonin exhibited sex-specificity (higher levels in males under long photoperiods, while females were unaffected by photoperiod). The higher melatonin levels in females correlated with more favorable lipid profiles and insulin sensitivity and showed a negative correlation with final body weight, suggesting a potential role in protecting females from metabolic disorders [42]. Regarding species specificity, mature female tilapia exhibit significantly higher hepatic melatonin concentrations and gonadal melatonin receptor mRNA levels compared to males. Distinct sex differences exist in melatonin concentrations within the tryptophan metabolic pathway. Exogenous melatonin administration improves glucose tolerance more significantly in female tilapia and can shift feeding behavior, growth phenotypes, and adipocyte area in male tilapia to more closely resemble those of females [43]. In terms of reproductive hormone regulation, the effect of melatonin on male gonadal hormones exhibits strict age-dependency. Only prepubertal rats showed significant decreases in plasma testosterone, LH, and FSH levels following melatonin injection, with no significant changes observed in pubertal or adult rats. Similarly, sex hormone synthesis in adult males shows no clear response to melatonin. In contrast, long-term daily melatonin supplementation (over 4 months) in women led to significant decreases in LH, estradiol, and progesterone levels, with some individuals even experiencing ovulation suppression. This confirms that prolonged exogenous melatonin exposure can inhibit ovarian steroidogenesis and secretion in females [44,45]. Collectively, these sex differences elucidate the sex-specific regulatory patterns of melatonin in both physiological and pathological processes, providing a theoretical basis for gender-targeted therapies in related diseases.

## 3. Melatonin and Mitochondrial Function

### 3.1. Melatonin in Maintaining Mitochondrial Membrane Potential

**Figure 2:**
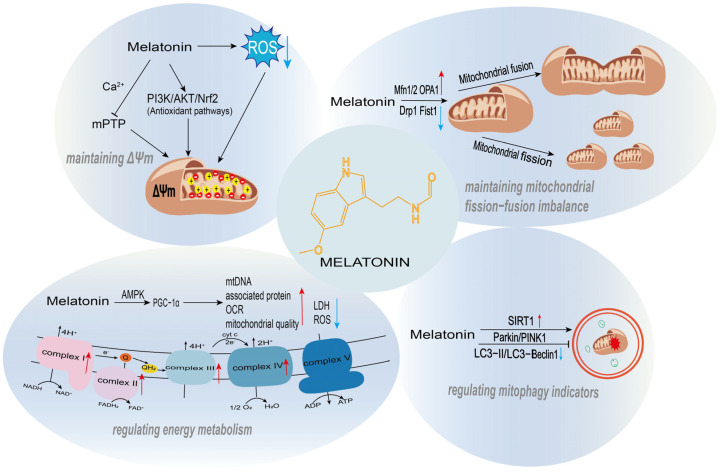
The regulatory roles of melatonin in mitochondrial function. Melatonin supports mitochondrial homeostasis through multiple mechanisms, including the maintenance of mitochondrial membrane potential, regulation of mitochondrial quality control, promotion of mitochondrial biogenesis, and modulation of energy metabolism.

Melatonin plays a crucial protective role in maintaining mitochondrial membrane potential (ΔΨm), one of its core mechanisms for regulating mitochondrial function (Figure 2). ΔΨm is fundamental for sustaining cellular energy metabolism and ion homeostasis; its collapse leads to calcium overload, ROS burst, and the initiation of apoptosis. ROS, as byproducts of the mitochondrial electron transport chain (ETC), trigger lipid peroxidation, calcium overload, and depolarization of ΔΨm when overproduced, ultimately inducing cell death. Melatonin stabilizes membrane potential by directly scavenging ROS, modulating the mitochondrial permeability transition pore (mPTP), and activating antioxidant signaling pathways, thereby preventing these detrimental events. Its high lipophilicity allows it to readily cross mitochondrial membranes and accumulate at high concentrations within the organelle, enabling immediate neutralization of ROS and mitigation of the associated damage.

Melatonin is a well-established potent free radical scavenger (directly neutralizing various ROS/RNS) through a non-receptor-mediated mechanism. It reacts with multiple reactive oxygen and nitrogen species, reducing their damage to mitochondrial membranes and functional components such as proteins and lipids. This direct scavenging action alleviates intra-mitochondrial oxidative stress, thereby preventing ROS-induced depolarization or collapse of ΔΨm. In a high glucose-induced oxidative stress model of Schwann cells, melatonin significantly suppressed ROS generation and restored ΔΨm. JC-1 probe assays revealed that high glucose reduced ΔΨm, while treatment with 0.5–10 μM melatonin partially reversed this depolarization. Concurrently, melatonin inhibited the mitochondrial-associated apoptotic pathway by regulating Bcl-2 family proteins (upregulating Bcl-2 and Bcl-xL, downregulating p-BAD and Puma), further indicating that its ΔΨm-stabilizing function is closely linked to its anti-apoptotic effects [46]. Hyun et al. demonstrated that cadmium (Cd) exposure induces intracellular ROS accumulation and mitochondrial dysfunction. Melatonin ameliorated these effects by upregulating mitochondrial STAT3 (mito-STAT3) levels and modulating the ETC component GRIM-19, thereby reducing ROS-mediated damage, improving mitochondrial function, and inhibiting cell death [47].

Aberrant opening of the mPTP is a major mechanism leading to ΔΨm collapse, often triggered by oxidative stress, calcium overload, or ROS. Melatonin directly inhibits mPTP opening, preventing calcium efflux and membrane depolarization, thus preserving membrane potential. In mouse and cellular models of hepatic ischemia-reperfusion (I/R) injury, melatonin pretreatment markedly attenuated I/R-induced mitochondrial ROS surge, ΔΨm loss, and functional impairment. This protection was mediated through suppression of the mitochondrial inner membrane protein PGAM5, whose activated form promotes mPTP opening. Melatonin administration inhibited this process and prevented hepatocyte death by blocking PGAM5 [48]. Studies have confirmed that melatonin and its metabolites (C3-OHM, AFMK) protect mitochondria by stabilizing them in a transient protective mode (t-MPT). In the t-MPT state, mitochondria expel excessive toxic substances while preserving ΔΨm to sustain cellular energy supply, thus avoiding apoptosis triggered by ΔΨm loss. Among these metabolites, C3-OHM exhibited a particularly pronounced effect in stabilizing t-MPT and protecting ΔΨm, while AFMK enhanced this protective action with antioxidant assistance [49]. Long-term melatonin administration reduced the sensitivity of mPTP to Ca^2+^, enhanced mitochondrial calcium retention capacity, alleviated swelling, and maintained ΔΨm stability. In an isoproterenol (ISO)-induced acute heart failure model in aged rats, melatonin reversed abnormal mPTP opening and ΔΨm loss, mitigated myocardial injury, and ultimately enhanced cardiomyocyte resistance to damage [50].

Activating antioxidant signaling pathways maintains ΔΨm homeostasis through a multi-level, network-like regulatory mechanism. The core logic lies in blocking oxidative stress-induced damage to membrane integrity, ion transport, and mitochondrial energy metabolism, while reinforcing protective effects through crosstalk between pathways. In a paraquat (PQ)-induced PD rat model, PQ exposure caused damage to dopaminergic neurons in the substantia nigra, exacerbated oxidative stress, and induced mitochondrial dysfunction, leading to ΔΨm instability. Melatonin exerted neuroprotective effects by activating the PI3K/AKT/Nrf2 antioxidant signaling pathway—upregulating downstream antioxidant molecules such as HO-1 and NQO1 to clear excess ROS, and enhancing the expression of mitochondrial complex-related genes NDUFS3 and SDHA to improve mitochondrial function, thereby stabilizing ΔΨm. This ultimately reversed PQ-induced reductions in tyrosine hydroxylase (TH)-positive neurons and TH protein expression, alleviating PD-like pathological damage [51]. A systematic study involving granulosa cells from patients with PCOS, a dihydrotestosterone (DHT)-induced PCOS-like mouse model, and the human granulosa cell line (KGN cells) demonstrated that melatonin specifically upregulates the NAD^+^-dependent deacetylase SIRT1, which serves as a key mediator for activating the PDK1/Akt signaling pathway. Knockdown of SIRT1 mRNA completely abolished melatonin’s activating effect on PDK1/Akt, confirming SIRT1 as the upstream core molecule of this regulatory pathway [52]. As a mitochondria-targeting molecule, melatonin protected hippocampal HT22 cells from oxygen-glucose deprivation/reperfusion (OGD/R) injury by upregulating SIRT3 and PGC-1α expression. SIRT3 enhances antioxidant capacity by deacetylating and activating SOD2, while PGC-1α promotes mitochondrial biogenesis and network remodeling, maintaining the activity of mtDNA-encoded respiratory chain complexes I, III, and IV, thereby providing a metabolic foundation for ΔΨm stability. Simultaneously, melatonin inhibits excessive mPTP opening, preventing mitochondrial calcium overload, membrane rupture, and mtDNA release, thereby avoiding mtDNA-induced activation of the cGAS-STING pathway and subsequent “mitochondrial inflammation.” It also modulates the timing of FGF-21 release to support stress adaptation. This selective, multi-faceted protection synergistically maintains ΔΨm stability, mitigates oxidative and inflammatory damage, and thereby safeguards cells against ischemic brain injury [53].

Increasing experimental evidence indicates that melatonin not only protects mitochondria from oxidative injury but can also directly enhance mitochondrial respiratory function. In obese and diabetic rodent models, chronic melatonin administration significantly increased the respiratory control ratio (RCR), ADP/O ratio, and ATP production in brown adipose tissue mitochondria, indicating an improvement in oxidative phosphorylation efficiency and coupling status [54]. Consistent with this, studies in senescent human granulosa-like cells have demonstrated that melatonin treatment elevates both basal and maximal mitochondrial oxygen consumption rate (OCR), accompanied by enhanced ATP generation and upregulation of oxidative phosphorylation complex proteins, thereby reflecting a global improvement in mitochondrial bioenergetic capacity [55]. Mechanistically, these effects appear to be mediated, at least in part, through activation of the SIRT1–PGC-1α axis and stimulation of mitochondrial biogenesis, which promotes renewal of functional mitochondrial networks and supports sustained respiratory competence [56]. Collectively, these findings confirm that melatonin exerts a positive regulatory influence on mitochondrial respiration.

### 3.2. Melatonin Regulation on Mitochondrial Quality Control

In recent years, substantial *in vivo* and *in vitro* experimental evidence has demonstrated that melatonin can exert its effects through dual modulation of mitochondrial dynamics: on one hand, it suppresses excessive mitochondrial fission, and on the other hand, it upregulates the expression or activity of mitochondrial fusion-related proteins (Mfn1/2, OPA1). These two mechanisms work in concert to restore or maintain the structural integrity and functional stability of the mitochondrial network (Figure 2).

As a molecule with established neuroprotective potential, melatonin can effectively counteract Cd-induced mitochondrial dynamic imbalance and functional impairment through precise targeted regulatory mechanisms. Cd exposure leads to cytosolic Ca^2+^ overload, which specifically drives the translocation of the key mitochondrial fission protein DRP1 from the cytoplasm to the mitochondrial membrane. This disrupts the dynamic balance between mitochondrial fission and fusion, resulting in the rapid transformation of mitochondria from a normal tubular network into a fragmented, punctate morphology. Consequently, this triggers secondary dysfunctions such as excessive ROS production, reduced ATP synthesis, loss of ΔΨm, and decreased cardiolipin content. Pretreatment with melatonin efficiently inhibits Cd-induced Ca^2+^ elevation and directly blocks DRP1 translocation to mitochondria. This intervention not only restores mitochondrial morphology by reversing fragmentation but also concurrently improves mitochondrial functional stability [57]. Furthermore, under conditions of obesity and metabolic stress, melatonin specifically corrects the unique mitochondrial fission/fusion imbalance in skeletal muscle, where fission capacity is suppressed (reduced Fis1/DRP1) and fusion activity is aberrantly enhanced (elevated OPA1/Mfn2). By upregulating fission markers and downregulating fusion markers, melatonin restores the DRP1/Mfn2 ratio, thereby re-establishing mitochondrial dynamic cycling and enabling quality control through SIRT1-modulated autophagy. This mechanistic correction underlies melatonin’s ability to repair mitochondrial function, reduce intramuscular lipid deposition, and ameliorate muscle fiber atrophy, ultimately enhancing structural integrity and metabolic flexibility [58]. In an oxidative damage model of ovarian granulosa cells (GCs), melatonin rectifies mitochondrial dynamic imbalance by regulating the core circadian clock gene Clock—downregulating the abnormally elevated expression of fission-related proteins (DRP1 and Fis1) while upregulating the reduced levels of fusion-related proteins (Mfn1 and OPA1). Simultaneously, it suppresses excessive mitophagy, repairs mitochondrial cristae integrity, inhibits granulosa cell pyroptosis, and ultimately improves oocyte developmental potential [59]. In a rat model of peripheral nerve injury (PNI) and an *in vitro* model of TBHP-induced RSC96 cell damage, melatonin significantly upregulated the expression of Parkin and PINK1, activating selective mitophagy. Clearing damaged mitochondria, it reduces ROS generation, maintains ΔΨm stability, and ensures autophagic flux. Additionally, it inhibits the expression of mitochondrial apoptosis-related proteins such as Bax and cleaved-caspase 3. These actions protect cell survival *in vitro* and promote remyelination while reducing collagen fiber proliferation around the injured tissue *in vivo*, ultimately facilitating peripheral nerve repair [60].

Melatonin demonstrates context-dependent regulatory effects on mitophagy. Under certain pathological conditions or cellular microenvironments, it activates Parkin/PINK1-dependent selective mitophagy to clear damaged mitochondria. Conversely, in other specific injury scenarios, it suppresses the overactivation of Parkin/PINK1-mediated mitophagy, thereby preventing cellular energy crises caused by excessive mitochondrial loss (Figure 2). In a CoCl_2_-induced hypoxia damage model using SH-SY5Y cells and a photothrombosis-induced ischemic stroke mouse model, melatonin significantly downregulated overactivated mitophagy markers. It reduced the expression levels of key mitophagy regulators PINK1 and Parkin, decreased the LC3-II/LC3-I ratio, inhibited the co-localization of the mitochondrial marker Tom20 with LC3, and directly reduced mitophagy events as observed by transmission electron microscopy (TEM). Furthermore, it reversed the decrease in mtDNA copy number in the stroke model and markedly improved cognitive deficits while reducing neuronal loss in the hippocampus and cortical regions of stroke mice [61]. In granulosa cells from PCOS patients, DHT-treated KGN cells, and a DHT-induced PCOS mouse model, melatonin specifically upregulated SIRT1 expression. This led to significant suppression of the overexpression of PINK1 and Parkin proteins, downregulation of autophagy markers (Beclin1 and LC3B-II), and an increase in the autophagy substrate p62. Consequently, it effectively inhibited excessive PINK1/Parkin-mediated mitophagy [62]. This regulatory action restored mitochondrial function, reduced mitochondrial loss, ameliorated PCOS-related phenotypes such as hormonal disturbances and polycystic ovarian morphology, and suppressed cell apoptosis.

### 3.3. Melatonin Promotes Mitochondrial Biogenesis and Energy Metabolism Remodeling

Melatonin regulates mitochondrial biogenesis and energy metabolism through a complete molecular pathway characterized by upstream signal sensing, core transcriptional mediation, and functional phenotypic output. It utilizes AMPK and SIRT1/SIRT3 as upstream regulatory hubs, leading to the modification and activation of PGC-1α, which then initiates a core transcriptional axis. This cascade ultimately enhances mitochondrial biogenesis and optimizes energy metabolism (Figure 2).

By activating the AMPK signaling pathway, melatonin directly upregulates the transcription and activity of PGC-1α, establishing the melatonin-PGC-1α axis. This axis serves as a core regulatory pathway mediating myocardial mitochondrial biogenesis and functional recovery. Specifically, through the activation of AMPK phosphorylation, melatonin directly drives the restoration of PGC-1α expression, subsequently regulating the transcription of downstream target genes, promoting mtDNA replication and the synthesis of mitochondrial-related proteins. This effectively restores the reduction in mitochondrial mass induced by hypoxia/reoxygenation (H/R) injury, facilitating mitochondrial biogenesis [63]. Concurrently, melatonin upregulates the expression of PGC-1α in mesenchymal stem cells (MSCs) via a receptor-dependent pathway. This enhances the activities of mitochondrial complexes I and IV, improves the oxygen consumption rate (OCR), and ameliorates energy metabolism under ischemic injury conditions. This axis further boosts the proliferative capacity of MSCs by upregulating cell cycle proteins (such as cyclin D1 and CDK4), promoting their migration and invasion, and increasing the secretion of angiogenic factors such as VEGF, FGF, and HGF [64].

In both female and male Zücker diabetic fatty (ZDF) rats and lean (ZL) rats, melatonin effectively improves mitochondrial energy metabolism in the vastus lateralis (VL) muscle. It reduces state 4 respiration associated with proton leak and restores or increases the respiratory control ratio (RCR) and phosphorylation coefficient (ADP/O ratio), thereby enhancing the efficiency of mitochondrial oxidative phosphorylation coupling. Simultaneously, melatonin activates the activities of respiratory chain complexes I, III, and IV, and reverses the significant downregulation of fatty acid oxidation (FAO) observed in ZDF rats, shifting substrate utilization toward a fatty acid-dependent profile. These effects depend on the activation of the NRF2/RCAN/MEF2 pathway, ultimately repairing obesity- and diabetes-related mitochondrial energy metabolism disorders [65].

*In vitro* experiments reveal that C2C12 myoblasts treated with melatonin exhibit significantly elevated OCR, including increased basal and maximal respiration rates, alongside augmented mitochondrial mass, elevated mtDNA content, and enhanced ΔΨm. The activities of mitochondrial respiratory chain complexes I, II, and IV are markedly increased, resulting in significantly higher ATP production. Furthermore, genes related to mitochondrial complex assembly, oxidative phosphorylation, and ATP synthesis are upregulated at the transcriptional level, while lactate dehydrogenase (LDH) activity and ROS levels are reduced. GSEA also demonstrates significant enrichment of gene sets associated with mitochondrial complex I assembly, mitochondrial ATP synthesis coupled electron transport, and oxidative phosphorylation in the melatonin-treated group [66]. Under metabolic stress conditions, melatonin promotes mitochondrial biogenesis-related regulation by upregulating Pgc1-α and Tfam, while enhancing the activities of β-hydroxyacyl-CoA dehydrogenase and citrate synthase to optimize β-oxidation. This improves antioxidant capacity, enabling more efficient substrate oxidation and energy supply within mitochondria. These findings provide experimental support for the role of melatonin as a metabolic modulator [67].

Mitochondria, as the core organelles regulating energy metabolism and redox homeostasis in the brain, exhibit distinct functional heterogeneity across different brain cell types and tissue regions. This inherent diversity directly modulates the injury mechanisms and repair capacities of various cell populations during the progression of neurodegenerative diseases. Melatonin, a neuroprotective agent with inherent mitochondrial-targeting properties, has been extensively verified through numerous studies to exert its protective effects via multiple pathways—including stabilizing mitochondrial membrane potential, regulating electron transport chain activity, enhancing the clearance of oxidative stress products, and maintaining mitochondrial dynamic balance. Notably, the protective efficacy of melatonin displays remarkable specificity, which is closely associated with the functional differences among distinct brain cell types. Table 2 summarizes the aforementioned cell-type-specific differences in melatonin’s protective effects.

### 3.4. The Relationship Between Mitochondria and NDs

Mitochondrial dysfunction is a pivotal pathological link in the onset and progression of NDs, characterized by multi-dimensional progression and mutual amplification of related pathological processes. Impairment of the mitochondrial respiratory chain and energy metabolism serves as an early core event in NDs. For instance, significantly reduced activity of Complex IV has been observed in the cerebral cortex of AD patients as early as the initial stages [68]. Similarly, deficiency of Complex I in the substantia nigra of PD patients has been consistently validated [69]. Subsequent studies further confirmed a marked accumulation of mtDNA deletions within nigral neurons in PD [70]. These findings collectively indicate that energy deficits and mtDNA damage can act as pathological amplifiers, driving neuronal functional decline. Concurrently, ROS derived from mitochondria not only cause cellular damage but also form positive feedback loops with toxic proteins such as Aβ, tau, and α-syn. For example, in AD models, Aβ interacts with the mitochondrial dynamin-related protein DRP1, leading to mitochondrial fragmentation and enhanced oxidative stress [71]. In PD models, α-syn directly inhibits Complex I activity, triggering increased ROS production [72]. This suggests that mitochondria serve as both the source of damage and the target of toxic proteins. Furthermore, disruption in the mitochondrial quality control system—comprising dynamics (fission/fusion) and mitophagy—can directly drive neurodegeneration. Specifically, the PINK1/Parkin pathway, associated with PD, mediates the selective clearance of damaged mitochondria [73]. Mutations in PINK1 are linked to inherited forms of PD [74], and loss of Mfn2 has been shown to result in neuronal degenerative damage [75], indicating that this process is not merely an epiphenomenon of pathology.

While different NDs exhibit significant clinical and molecular pathological diversity, growing experimental evidence underscores that these diseases share a central pathological axis: mitochondrial dysfunction. Mitochondria are essential for critical neuronal functions including ATP generation, calcium homeostasis maintenance, and ROS regulation. Consequently, the disruption of mitochondrial homeostasis is not merely a concomitant event in NDs but likely constitutes a significant driving force in pathogenesis. Meanwhile, melatonin, due to its mitochondrial localization, antioxidant properties, and signaling regulatory effects, is recognized as having unique potential for mitochondrial protection.

## 4. The Role of Melatonin in Neurodegenerative Diseases

In the context of aging and the development of neurodegenerative disorders, two core pathological pathways have become increasingly evident: on one hand, there is a progressive decline in the endogenous synthesis and secretion of melatonin; on the other, there is a systematic deterioration of mitochondrial function in neurons. These two processes are not isolated but interact in a cascade manner, collectively driving the progression of neurodegenerative pathology. Melatonin mediates a variety of receptor-dependent physiological functions *in vivo* through two high-affinity G-protein coupled receptors, namely MT1 (MTNR1A) and MT2 (MTNR1B). These receptors are widely expressed in the central nervous system and peripheral tissues. By coupling to G proteins such as Gi/o and Gq/11, they inhibit cAMP production, modulate intracellular Ca^2+^ levels, and activate downstream signaling pathways including ERK and PI3K/Akt, thereby influencing circadian rhythms, sleep–wake cycles, neuronal activity, vascular tone, and immune responses. Simultaneously, melatonin exhibits prominent receptor-independent functions. As a lipophilic indoleamine, it readily crosses cellular membranes and the blood-brain barrier, directly scavenging ROS/RNS radicals. Additionally, melatonin alleviates cellular oxidative stress by enhancing antioxidant enzyme activity and suppressing pro-oxidant enzyme activity. Furthermore, within mitochondria, melatonin helps maintain ΔΨm, inhibits the opening of the mPTP, and attenuates apoptotic signaling, thereby exerting anti-inflammatory, anti-apoptotic, and cytoprotective effects [76,77,78].

As previously described, melatonin levels in various tissues decline significantly with advancing age and in the presence of multiple systemic or localized diseases. Concurrently, mitochondria exhibit a range of aging-related phenotypes, including diminished respiratory capacity, loss of ΔΨm, accumulation of mtDNA damage, and impairments in mitochondrial dynamics and quality control. The decline in melatonin is not merely a bystander phenomenon; its deficiency weakens multiple mitochondrial protective pathways, thereby amplifying mitochondrial damage and establishing a vicious cycle. Conversely, melatonin supplementation has been shown in numerous animal and cellular studies to partially reverse such damage. In elderly individuals, particularly frail older adults, nocturnal and circadian melatonin peaks are markedly reduced. Moreover, cerebrospinal fluid (CSF) melatonin levels decrease markedly with age. In patients with cognitive impairment such as AD, CSF melatonin levels are significantly lower than in age-matched non-demented older subjects, with an even more pronounced reduction observed in AD patients carrying two APOE4 alleles [79]. This decline is primarily associated with reduced secretory function of the pineal gland. While a single study has suggested that the choroid plexus may also synthesize melatonin, this finding requires further validation. The resulting insufficient endogenous melatonin supply in the brain may exacerbate cognitive decline [80]. In the context of mitochondrial diseases associated with the mtDNA 3243A>G mutation and age-related neuronal mitochondrial damage, melatonin has been shown to scavenge mitochondrial ROS, restore ΔΨm and respiratory chain/fusion functions, inhibit excessive MAPK activation, and reduce mitochondrial-associated apoptosis, thereby protecting neuronal mitochondrial function [81]. In studies involving aged mice, melatonin treatment improved 24-h memory performance in novel object recognition (NOR) and novel location recognition (NLR) tasks. This cognitive enhancement was linked to the modulation of mitochondrial function in the prefrontal cortex (PFC) and hippocampus, along with enhanced clearance of various free radicals [82]. These findings suggest that melatonin can partially counteract age-related cognitive decline and the underlying mitochondrial dysfunction.

With advancing age and the progression of various chronic conditions, the evidence presented consistently demonstrates a parallel decline in both systemic and tissue-specific melatonin levels and the deterioration of neuronal mitochondrial homeostasis—manifested by impairments in respiration, membrane potential, quality control, and biogenesis. Translating these general/mechanistic insights into specific disease contexts reveals that melatonin not only serves as a measurable biomarker of neurological vulnerability but also represents a potential therapeutic target for intervention.

### 4.1. Melatonin and Alzheimer’s Disease (AD)

**Figure 3:**
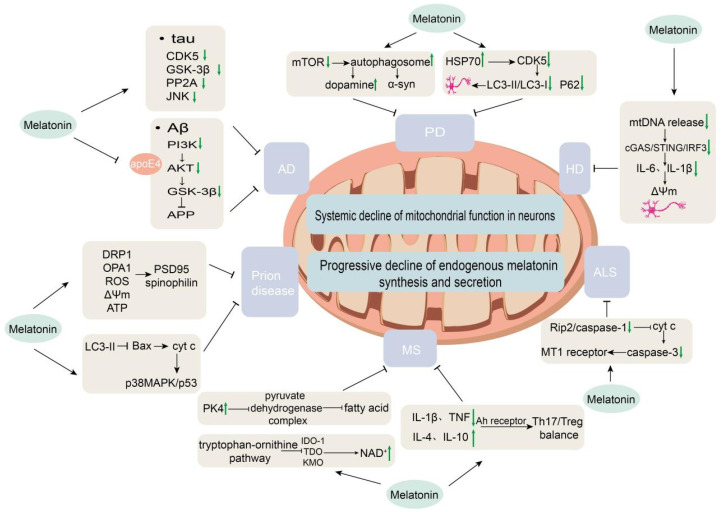
Mechanisms of melatonin regulation in neurodegenerative diseases. This schematic illustrates the disease-specific regulatory actions of melatonin in Alzheimer’s disease (AD), Parkinson’s disease (PD), Huntington’s disease (HD), amyotrophic lateral sclerosis (ALS), multiple sclerosis (MS), and prion diseases.

AD is a chronic neurodegenerative disorder and the leading cause of dementia, projected to affect over 150 million people worldwide by 2050 [83]. As one of the most pressing neurodegenerative challenges of our time, AD is characterized by progressive cognitive decline, β-amyloid (Aβ) plaque deposition, neurofibrillary tangles (NFTs) composed of hyperphosphorylated tau protein, oxidative stress, neuroinflammation, mitochondrial dysfunction, and circadian rhythm disturbances. Mitochondrial dysfunction is a core pathological hallmark of AD, contributing to neuronal metabolic instability and driving disease progression [84]. Acute hypoxia, as a potential risk factor for AD, exacerbates mitochondrial respiratory deficits and morphological abnormalities in preclinical models. Impairments in melatonin secretion and signaling are directly linked to mitochondrial dysfunction, inflammation, and circadian disruption, reflecting a bidirectional relationship between mitochondrial failure and melatonin deficiency [85]. These pathological features significantly overlap with the known molecular targets of melatonin. Consequently, melatonin may serve not only as a potential biomarker but also as a multi-target therapeutic node, intervening in AD pathogenesis through its direct antioxidant activity, mitochondrial stabilization, inhibition of protein aggregation, modulation of autophagy and proteostasis, and restoration of circadian rhythms and sleep (Figure 3).

Melatonin exerts its effects via activation of the PI3K/Akt pathway through MT1 and MT2 receptors, leading to Akt-mediated phosphorylation and inhibition of GSK-3β (at Ser9), thereby suppressing amyloidogenic APP processing and reducing Aβ production. Simultaneously, it exerts anti-apoptotic effects by downregulating caspase-3 and upregulating Bcl-2 [86]. Furthermore, melatonin counteracts the synergistic neurotoxicity of Aβ and apolipoprotein E4 (apoE4) by binding to apoE4 [87]. In streptozotocin-induced AD model rats, melatonin inhibits Aβ production through downregulation of β-/γ-secretase and upregulation of α-secretase, while also directly binding to and disaggregating Aβ fibrils to block aggregation [88]. In SH-SY5Y cells, it reduces Aβ synthesis by inhibiting the transcription and activity of β-/γ-secretase; alleviates Aβ toxicity by enhancing mitochondrial function and reducing oxidative stress; maintains intracellular trafficking through microtubule-associated proteins and Rho GTPase pathways; and suppresses NMDA receptor-mediated excitotoxicity [89]. In high-fat diet-induced AD-like models, melatonin improves synaptic dysfunction by downregulating β-secretase and reducing Aβ generation [90].

Tau is a microtubule-associated protein that stabilizes the cytoskeleton and promotes microtubule assembly. In AD brains, 43–55 phosphorylation sites on tau have been identified [91]. Melatonin exerts multi-nodal regulation on tau phosphorylation, providing layered protection through site coverage and pathway redundancy. Melatonin upregulates miR-504-3p, which binds to the 3′-UTR of p39—an activator of the tau kinase CDK5—thereby suppressing p39 expression, reducing CDK5 activity, decreasing AD-relevant tau hyperphosphorylation, and attenuating NFT formation and neuronal loss in the cortex and hippocampus of hTau mice, with efficacy observed in both early and late intervention stages [92]. In AD pathology, Aβ1-42 aggregation triggers tau abnormalities; melatonin inhibits Aβ1-42-induced hyperphosphorylation of tau at Ser404 and Ser413 via the PI3K/Akt/GSK-3β pathway, reducing tau-mediated neurotoxicity [93]. Under ischemic injury, melatonin also maintains levels of the PP2A regulatory subunit B, activating PP2A-mediated tau dephosphorylation and preventing axonal degeneration and neuronal damage [94]. MT1 and MT2 receptor signaling influences tau phosphorylation: receptor knockout elevates cAMP levels, reduces miR-125b-5p expression, leads to PME-1 overexpression and decreased PP2A activity, and results in tau hyperphosphorylation at Ser404, Ser262, and Thr231. Ghost-like NFTs are observed in MT2KO and DKO mice, while argentophilic deposits appear in MT1KO mice, with Ser262 phosphorylation showing the strongest association with NFT formation [95]. Additionally, melatonin activates nuclear SIRT1 and mitochondrial SIRT3. SIRT1 deacetylates PGC-1α and NF-κB to indirectly reduce the activity of tau-related kinases such as GSK-3β, while SIRT3 deacetylates MnSOD to lower mitochondrial ROS, thereby inhibiting ROS-mediated JNK activation and abnormal tau modification. Inhibition of SIRT1/SIRT3 partially blocks these protective effects [96].

In streptozotocin-induced AD model rats, melatonin directly binds to and disaggregates Aβ fibrils, blocking plaque formation [88]. High-fat diet-induced AD-like models demonstrate that melatonin activates the SIRT1/Nrf2/HO-1 signaling pathway to reduce Aβ generation and synaptic dysfunction; moreover, this protective effect exhibits transgenerational persistence, suppressing abnormal Aβ accumulation in the brains of offspring [90]. In hTau mice, melatonin reduces pathological tau hyperphosphorylation and neurofibrillary tangle formation in both early and late intervention stages [92]. A meta-analysis incorporating eight randomized controlled trials (518 participants) confirmed that melatonin significantly improves cognitive function in adults with cognitive impairment, with more pronounced efficacy in patients with mild cognitive impairment (MCI)—a prodromal stage of AD. The optimal regimen involved evening administration (20:30–21:00) for 13–24 weeks, showing favorable safety with only mild gastrointestinal adverse events reported [97].

Current evidence evidence for the neuroprotective effects of melatonin in AD is currently derived mainly from preclinical studies using cellular and animal models. Existing clinical evidence largely focuses on the mild cognitive impairment stage, with studies being limited in sample size, short in follow-up duration, and high in heterogeneity—insufficient to support a clear disease-modifying effect. Lack of standardization across studies regarding dosage, timing of administration, and outcome measures increases the difficulty of evidence integration and interpretation. Additionally, the mechanisms underlying melatonin’s combined effects with other therapies, as well as its long-term safety, require further validation through large-scale randomized controlled trials. Hence, although mechanistic research and early clinical explorations demonstrate potential, the true clinical value of melatonin in AD treatment still awaits support from higher-quality evidence.

### 4.2. Melatonin and Parkinson’s Disease (PD)

PD is a rapidly progressive neurodegenerative disorder that affects approximately 1% of individuals over 60 years of age, with prevalence reaching up to 4% in the elderly population. It is characterized by the loss of dopaminergic neurons in the substantia nigra pars compacta, leading to motor symptoms (bradykinesia, tremor, rigidity) as well as non-motor manifestations including sleep disturbances and circadian dysregulation [98]. Mitochondrial dysfunction, elevated oxidative stress markers, α-syn aggregation, and neuroinflammation are closely linked to PD pathogenesis. In PD, melatonin secretion is significantly disrupted, contributing to circadian desynchrony and symptom exacerbation. Patients exhibit reduced nocturnal melatonin production, circadian rhythm disturbances, and abnormalities in the sleep-wake cycle, which correlate with non-motor symptoms such as REM sleep behavior disorder (RBD) and daytime sleepiness. Relevant clinical studies and meta-analyses indicate that oral melatonin supplementation can improve subjective sleep quality in PD patients [99,100].

The pathological core of PD lies in the loss of dopaminergic neurons in the substantia nigra pars compacta, which is closely associated with mitochondrial dysfunction. In the substantia nigra of PD patients, mRNA expression of the melatonin receptor MT1 is significantly reduced. MT1 deficiency promotes mitochondrial fission and dysfunction by inhibiting protein kinase A (PKA)-mediated phosphorylation of DRP1 at Ser637 while enhancing ERK1/2-mediated phosphorylation at Ser616. This also impairs autophagic flux and aggravates α-syn aggregation [101].

Melatonin is a pleiotropic neuroprotective agent that intervenes in mitochondrial dysfunction and oxidative stress in PD through multi-target mechanisms. The complexity of melatonin signaling leads to considerable variability in the effects of different melatonin receptor agonists in PD [102]. A clinical double-blind trial demonstrated that PD patients treated with melatonin showed significantly reduced levels of plasma lipid peroxides, nitric oxide metabolites, and protein carbonyls, along with enhanced catalase activity. Moreover, platelet mitochondrial complex I activity and respiratory control ratio were restored to levels comparable to healthy controls, with no alteration in membrane fluidity, directly improving mitochondrial bioenergetic function [103]. At the mechanistic level, studies using paraquat-induced PD models revealed that these effects are mediated by the PI3K/AKT/Nrf2 signaling cascade. Melatonin dose-dependently upregulates phosphorylated AKT and Nrf2 expression, subsequently inducing the antioxidant enzymes heme oxygenase-1 (HO-1) and NAD(P)H quinone oxidoreductase 1 (NQO1), while protecting key mitochondrial respiratory chain components NDUFS3 and SDHA. Activation of this pathway is central to melatonin’s ability to attenuate ROS burst, protect TH-positive dopaminergic neurons, and counteract oxidative damage [51].

In PD treatment, melatonin exhibits a cell-type and pathology-dependent dual regulatory mechanism: it can promote neural repair by inducing autophagy while protecting existing neurons by suppressing excessive autophagy, highlighting its pleiotropic neuroprotective value (Figure 3). Specifically, during the differentiation of adipose-derived mesenchymal stem cells (AD-MSCs) into dopaminergic neurons, melatonin activates autophagy by downregulating the mTOR pathway, significantly increasing autophagosome formation, promoting α-syn clearance, and enhancing dopamine secretion [104]. Conversely, in MPP+-induced PD mouse models, melatonin upregulates heat shock protein HSP70 expression to inhibit CDK5-mediated excessive autophagy, reduces the LC3-II/LC3-I ratio and p62 accumulation, thereby protecting nigral dopamine transporter-positive neurons and improving motor function [105]. This seemingly contradictory regulation reflects the dual role of autophagy in PD pathology: moderate activation is required during stem cell differentiation to clear pathological protein aggregates, whereas excessive activation in mature neurons must be inhibited to prevent cell death. Melatonin alleviates neuroinflammation by downregulating pro-inflammatory cytokines and inhibiting microglial over-activation. In PD models, expression of the retinoic acid-related orphan nuclear receptor α (RORα) is significantly downregulated, accompanied by polarization of microglia toward the pro-inflammatory M1 phenotype, activation of disease-associated microglia (DAM), elevated pro-inflammatory cytokines (TNF-α, IL-1β, IL-6), loss of dopaminergic neurons, and impaired motor function. Melatonin intervention significantly upregulates RORα, which in turn modulates the STAT pathway to reduce pro-inflammatory cytokine secretion, suppress M1 and DAM phenotypes, and promote the anti-inflammatory M2 phenotype in microglia. Through these actions, melatonin protects TH-positive dopaminergic neurons in the substantia nigra and improves motor function in PD mouse models [106]. In paraquat-induced PD models, melatonin dose-dependently activates the PI3K/AKT/Nrf2 signaling pathway, preserves tyrosine hydroxylase-positive dopaminergic neurons, and counteracts oxidative damage—effects that are completely abolished by a PI3K-specific inhibitor [51].

Currently, evidence regarding the neuroprotective effects of melatonin in PD remains largely based on mechanistic studies and animal models, while clinical research has primarily focused on improving non-motor symptoms such as sleep and circadian rhythms. Evidence for disease-modifying effects is limited. Previous clinical trials have exhibited marked heterogeneity in sample size, treatment dosage, duration, and endpoint selection, and most are short-term studies, constraining the evaluation of long-term efficacy and safety. Furthermore, differences in disease stage, concomitant medications, and pathological heterogeneity among PD patients may influence melatonin responsiveness and complicate result interpretation. Therefore, large-scale, rigorously designed randomized controlled trials are still needed to validate the true clinical value of melatonin in the treatment of PD.

### 4.3. Melatonin and Huntington’s Disease (HD)

HD is an inherited neurodegenerative disorder caused by CAG repeat expansion in the HTT gene, leading to accumulation of mutant huntingtin protein (mHTT) and subsequent damage to neurons in the basal ganglia and cortex [107]. Melatonin, a key circadian regulator with well-defined mitochondrial protective, antioxidant, and immunomodulatory properties, has garnered renewed interest in recent HD research.

In HD, local melatonin synthesis within synaptic mitochondria is impaired as mHTT aggregates sequester the rate-limiting enzyme AA-NAT [108]. Critically, the expression and localization of mitochondrial MT1 receptors are reduced, hindering melatonin from effectively modulating mitochondrial respiration [109]. mHTT also disrupts mitochondrial function, leading to mtDNA release into the cytosol, which activates the cGAS/STING/IRF3 inflammatory pathway and caspase-1, thereby triggering neuroinflammation and neurodegeneration. HD patients additionally exhibit decreased melatonin levels. Using HD models, exogenous melatonin has been shown to suppress mtDNA release, downregulate cGAS/STING/IRF3 activation, reduce production of pro-inflammatory cytokines such as IL-6 and IL-1β, ameliorate mitochondrial oxidative stress, restore ΔΨm, and protect synapses and neurons from degeneration. Supporting evidence from AA-NAT knockout models further confirms melatonin’s pivotal role in maintaining mitochondrial homeostasis and attenuating inflammation [110]. Collectively, these findings suggest that melatonin ameliorates HD pathology by modulating mtDNA-mediated inflammatory responses, offering a potential therapeutic avenue for HD and related neurodegenerative disorders. Melatonin also stabilizes specific neurotransmitter levels, preserves neuronal morphology in the striatum, cortex, and cerebellum, and improves behaviors related to fine motor coordination in HD rat models, although it does not affect non-learning-associated behaviors or memory deficits [111] (Figure 3).

In HD, melatonin secretion exhibits distinct circadian disturbances. Studies indicate that in early-stage patients, the nocturnal rise of melatonin is delayed by approximately 1.5 h on average, resembling a delayed sleep-phase phenotype. This phase delay is not an isolated phenomenon but reflects disease progression--melatonin levels (including mean concentration, peak amplitude, and secretion magnitude) progressively decline with advancing disease, and reductions can be detected even at the presymptomatic stage. Notably, melatonin levels correlate inversely with the severity of motor impairment, suggesting its potential as a biomarker of disease progression. Early physiological studies reported delayed nocturnal melatonin onset in HD patients, with some cohorts showing reduced total plasma or salivary melatonin, supporting the view that both the rhythmicity and amplitude of melatonin secretion are impaired [112,113,114]. In HD, mHTT-induced reduction of mitochondrial MT1 prevents melatonin from effectively modulating mitochondrial metabolism. Combined with ligand deficiency due to impaired local synthesis, this dual defect contributes to mitochondrial dysfunction closely associated with the neurodegenerative process in HD. In transgenic HD mouse models, melatonin delays disease onset, extends lifespan, and reduces ventricular enlargement. It inhibits mHTT-induced caspase activation, prevents release of mitochondrial pro-apoptotic factors, and protects MT1 receptors from degradation. Further validation shows that knockdown of MT1 abolishes the protective effects of melatonin, whereas MT1 overexpression enhances cellular resistance. Moreover, MT1 receptor levels gradually decline in post-mortem human HD brain tissues with disease progression, confirming the link between MT1 loss and HD pathology [115]. An AA-NAT knockout mouse model, characterized by endogenous melatonin deficiency, further demonstrates exacerbated mitochondrial dysfunction and neuroinflammation. In these mice, increased mitochondrial oxidative stress, reduced mitochondrial membrane potential, and elevated mtDNA release were observed in the brain and primary cortical neurons. Cytosolic mtDNA activated the cGAS/STING/IRF3 pathway, stimulating the production of inflammatory cytokines [110]. A double-blind, randomized, placebo-controlled crossover trial involving 15 HD gene carriers with sleep disturbances reported that 5 mg fast-release melatonin administered for 4 weeks did not significantly improve sleep quality, cognitive function, neuropsychiatric symptoms, or motor function compared to placebo. However, melatonin was well tolerated without adverse events. The study acknowledged limitations such as small sample size and lack of objective sleep assessments, suggesting that future research should explore alternative dosages, individualized regimens, and stage-specific effects [116].

Although extensive cellular and animal model studies support the mitochondrial protective, antioxidant, and anti-inflammatory effects of melatonin in HD, and highlight the critical regulatory role of the melatonin-MT1 receptor axis, clinical evidence for HD patients remains very limited. Existing clinical cohorts are predominantly small-scale and cross-sectional in design, lacking randomized controlled intervention trials. Heterogeneity in sampling timing and disease stages also makes it difficult to establish a clear causal relationship between melatonin levels and clinical manifestations. Furthermore, factors such as the instability of melatonin, variability in its bioavailability, and region-specific alterations in receptor expression add complexity to result interpretation. Therefore, larger-scale, longitudinal, and mechanism-oriented clinical studies are required to validate the therapeutic potential of melatonin in HD.

### 4.4. Melatonin and Other Neurodegenerative Diseases

Melatonin is not merely a circadian rhythm-regulating hormone within the suprachiasmatic nucleus–pineal gland axis, but also a potent free radical scavenger, mitochondrial protector, and cellular homeostasis modulator. In recent years, beyond AD, PD, and HD, accumulating evidence points to the potential role of melatonin in a variety of other neurodegenerative disorders (Figure 3).

Amyotrophic lateral sclerosis (ALS) is a fatal neurodegenerative disease that primarily affects motor neurons in the brain and spinal cord, leading to progressive muscle atrophy and paralysis, with an average survival of 3–5 years after diagnosis. Approximately 90% of cases are sporadic and 10% familial. The core pathological mechanisms involve multifactorial damage to motor neurons, including TDP-43 protein aggregation, glutamate excitotoxicity, oxidative stress, mitochondrial dysfunction, and neuroinflammation [117]. Early in the disease course, ALS patients exhibit objective changes in sleep architecture, characterized by increased wakefulness and decreased deep sleep, with clinically significant sleep disturbances present in 66% of cases. Dysfunction of hypothalamic sleep-regulatory neuropeptide systems may represent a central mechanism, and these sleep abnormalities are significantly correlated with cognitive decline [118,119]. These symptoms are associated with disrupted melatonin secretion rhythms, suggesting that melatonin replacement or circadian realignment might alleviate symptom burden and indirectly influence disease progression. Mitochondrial dysfunction is a central pathological mechanism in ALS, contributing to motor neuron degeneration via oxidative stress, respiratory impairment, and cytochrome c release [117,120]. Exogenous melatonin has been shown to delay disease onset, improve motor function, and extend survival in SOD1G93A mice by inhibiting the Rip2/caspase-1 upstream signaling pathway, blocking mitochondrial cytochrome c release, reducing caspase-3 activation, and restoring downregulated MT1 receptor expression in ALS models, providing direct evidence for the melatonin-MT1 receptor axis as a therapeutic target in ALS [120]. However, the results are not entirely consistent: Dardiotis et al. reported that intraperitoneal injection of melatonin in G93A SOD1 mice not only failed to show protection but also shortened survival, aggravated motor neuron loss, and upregulated mutant SOD1 expression, thereby counteracting its antioxidant effects. This indicates that the efficacy of melatonin in ALS models is strictly dependent on dosage and model specificity [121]. Analysis of the Pooled Resource Open-Access ALS Clinical Trials (PRO-ACT) database showed that among 18 ALS patients using melatonin continuously compared to 1604 non-users, the melatonin group had a 76% reduced mortality risk and significantly slower decline in functional scores (sALSFRS) and pulmonary function (FVC) [122]. Nevertheless, current clinical evidence is limited to observational database analyses, lacking prospective randomized intervention studies. Preclinical findings are inconsistent and highly dose-dependent. No completed interventional randomized clinical trials have been reported.

Multiple sclerosis (MS) is the most common immune-mediated inflammatory demyelinating disease of the central nervous system, characterized pathologically by multifocal demyelinating lesions with oligodendrocyte loss, astrogliosis, and axonal injury. Clinically, it manifests with diverse and highly heterogeneous symptoms, commonly involving motor, sensory, and visual dysfunction, and can follow a relapsing-remitting or progressive course. The disease exhibits distinctive epidemiological features, such as higher incidence at higher latitudes and a female predominance, with both genetic and environmental factors contributing to its pathogenesis [123]. Mitochondrial dysfunction contributes to impaired energy metabolism and neuro-axonal injury in MS, though its phenotypic features are less distinct than in other neurodegenerative diseases [123]. In the experimental autoimmune encephalomyelitis (EAE) model, melatonin exerts protection through multiple pathways: it upregulates PDK4 expression, thereby inhibiting pyruvate dehydrogenase complex activity and potentially affecting fatty acid synthesis. Its anti-inflammatory and immunomodulatory actions are reflected in the downregulation of pro-inflammatory cytokines such as IL-1β and TNF, upregulation of anti-inflammatory cytokines including IL-4 and IL-10, and modulation of Th17/Treg balance via the aryl hydrocarbon receptor [124]. Moreover, melatonin intervenes in the tryptophan-kynurenine pathway by inhibiting key enzymes such as IDO-1, TDO, and KMO to reduce the accumulation of neurotoxic metabolites, and suppresses the overexpression of nicotinamide N-methyltransferase to elevate NAD^+^ levels and improve energy metabolism [125]. Observational studies indicate that untreated MS patients exhibit reduced 24-h urinary excretion of the melatonin metabolite 6-sulfatoxymelatonin (6-SMT) [126]. Clinical trials further confirm that immunomodulatory therapy significantly increases serum melatonin concentrations in patients with relapsing-remitting MS [127]. All clinical evidence is derived from observational studies, lacking prospective interventional trials with melatonin. The relationship between melatonin supplementation and disease progression has not yet been explored in humans.

Prion diseases are fatal neurodegenerative disorders caused by abnormal prion protein (PrP), lacking nucleic acids and consisting solely of protein, characterized by rapidly progressive dementia, 100% mortality, and absence of effective treatment [128]. In cellular models of prion disease, melatonin exerts neuroprotection via two core mechanisms. First, it modulates mitochondrial dynamics and homeostasis: exogenous melatonin pretreatment reverses PrP106-126-induced abnormal accumulation of the mitochondrial fission protein DRP1 and downregulation of the fusion protein OPA1, suppresses excessive mitochondrial fragmentation, improves perinuclear distribution, reduces excessive ROS production, and restores ATP levels and ΔΨm, thereby protecting synaptic markers such as PSD95 and spinophilin. Second, melatonin activates the autophagy-lysosomal pathway: it increases the autophagy marker LC3-II in a dose-dependent manner to clear damaged mitochondria, and blocks Bax translocation to mitochondria, cytochrome c release, and activation of the downstream p38MAPK/p53 apoptotic pathway. This protective effect can be specifically reversed by the autophagy inhibitor 3-MA or ATG5 siRNA [8,129].

In both cellular and animal models, melatonin delays prion-associated neuropathology and improves survival outcomes; however, clinical evidence remains limited, and large-scale randomized controlled trials are still lacking to establish its disease-modifying effects in human prion diseases.

The role of melatonin in neurodegenerative diseases is summarized in Table 3, including the types of diseases, mitochondrial targets, melatonin mechanisms, evidence (cellular/animal/human), and the pathways used in key studies.

## 5. Conclusions

The neuroprotective potential of melatonin in neurodegenerative diseases is well-established across preclinical and translational studies, positioning it as a promising multi-target therapeutic candidate. Its efficacy arises from a constellation of mitochondrial-centered mechanisms, including direct scavenging of ROS, stabilization of ΔΨm, regulation of autophagy-ubiquitin-mediated quality control, enhancement of oxidative phosphorylation complex activity, and promotion of mitochondrial biogenesis to restore energy homeostasis. These core actions have been consistently validated in experimental models of AD, PD, and HD.

Despite these strengths, both the current research landscape and its clinical translation warrant critical evaluation. The evidence base remains largely preclinical, resulting in significant translational barriers such as low predictive validity of animal models, small clinical trial sample sizes, lack of standardized dosing and treatment timing, and insufficient long-term safety data. Furthermore, potential sex differences in melatonin synthesis, metabolism, and biological effects may lead to heterogeneous responses to treatment and variable safety thresholds between male and female patients. However, most preclinical studies have not adequately accounted for this variable, and clinical trials often lack sex-stratified analysis. This gap further complicates the translation of basic research findings into clinical applications. Although systematic reviews indicate that melatonin is generally well tolerated with no unacceptable toxicity, even at higher doses for short-to-medium durations [130], caution remains essential for elderly patients with neurodegenerative diseases, who frequently experience polypharmacy. Melatonin may interact with various medications via metabolic pathways such as CYP1A2, potentially causing unpredictable shifts in plasma drug concentrations [131]. Additionally, risks such as enhanced bleeding when combined with anticoagulants or antiplatelet agents, and increased sedation and fall risk when co-administered with other sedative drugs. These safety considerations are especially critical in elderly patients with cognitive impairment and physical frailty and have been consistently emphasized in clinical practice [132]. Therefore, in designing clinical trials or implementing melatonin therapy for older adults with neurodegenerative diseases, it is imperative to systematically evaluate key safety endpoints such as drug-drug interactions, falls/fractures, and bleeding events, and to conduct individualized risk-benefit assessments.

Although some randomized controlled trials on melatonin have been conducted in AD and PD, focusing primarily on improving sleep quality or non-motor symptoms, clinical research targeting disease progression or cognitive/motor function improvement in AD/PD remains relatively limited compared to conditions such as ALS and MS. This limitation is reflected in several key challenges. First, the pathological mechanisms of AD and PD are highly complex and heterogeneous, involving multiple processes such as β-amyloid and tau pathology, α-synuclein aggregation, and neuroinflammation. The circadian-regulating and antioxidative effects of melatonin, as a single-target intervention, are difficult to translate into significant disease-modifying outcomes, posing high demands on trial design, endpoint selection, and statistical analysis. Second, existing clinical trials lack standardization in dosage and administration protocols. Most studies have employed low doses to address sleep issues, without systematically evaluating the effects of higher doses or long-term administration on neurodegenerative progression. Moreover, many studies are limited by small sample sizes, short follow-up periods, and primary endpoints centered on sleep scores rather than core cognitive or motor measures, thereby constraining robust efficacy evaluation. Third, difficulties in early diagnosis and disease-stage stratification further complicate clinical trials, as the potential neuroprotective effects of melatonin are more likely to manifest in early disease stages. However, the identification and recruitment of early-stage AD/PD patients remain challenging in themselves. Collectively, these design- and pathology-related hurdles impede large-scale, long-term clinical validation of melatonin as a disease-modifying therapy in AD and PD [133,134,135].

To bridge the gap between promising mechanistic evidence and proven clinical utility, future research must adopt a more integrated and rigorous approach. Mechanistic studies should advance beyond phenomenological observations to precise characterization of dose-response relationships, optimal treatment windows, and cell-type-specific signaling pathways within the neurovascular unit. Clinically, large, well-designed trials are urgently needed, incorporating stratified enrollment based on disease stage, genetics, circadian rhythm status, and concomitant medication profiles to identify responsive patient endophenotypes. Parallel efforts should focus on optimizing pharmacokinetics—comparing formulations, defining brain and mitochondrial bioavailability, and establishing dose schedules aligned with circadian biology and disease pathophysiology. Equally important is the development of standardized safety monitoring frameworks for elderly patients, emphasizing drug interactions, fall and bleeding risks, and cognitive side effects. In addition, real-world evidence from large registries and long-term cohort studies will be indispensable for evaluating sustained efficacy and detecting rare adverse events.

In summary, melatonin represents a mechanistically compelling candidate within the therapeutic landscape of neurodegenerative diseases. However, its path to routine clinical adoption must be approached with caution. Fully realizing its full neuroprotective potential will require the development of a robust and comprehensive clinical evidence base—one that demonstrates not only meaningful efficacy but also rigorous safety in the vulnerable patient populations it aims to serve.

## Figures and Tables

**Table 1:** Accumulation of Melatonin in Mitochondria.

Mechanism	Specific Methods
Passive Transmembrane Diffusion (Cellular Level)	The amphiphilic and lipophilic nature of melatonin allows it to traverse cellular membranes to enter cells and tissues [28]
Active Mitochondrial Uptake	The oligopeptide transporters PEPT1/2 (SLC15A1/2) located on mitochondrial membranes mediate the transmembrane uptake of melatonin [22] The transport mediated by PEPT1/2 facilitates the high-concentration enrichment of melatonin within mitochondria [23,24]
Endogenous Biosynthesis within Mitochondria	Mitochondria express key melatonin synthesis enzymes, AA-NAT and ASMT, enabling local production of melatonin directly within the organelles [25,26]
Non-Pineal Maintenance Mechanism	Following pinealectomy, tissue melatonin levels remain stable, suggesting that peripheral tissue and mitochondrial melatonin sources are independent of circulatory secretion [29]
Evolutionarily Conserved Enrichment Phenomenon	The enrichment of melatonin within mitochondria is considered an evolutionarily conserved strategy to enhance antioxidative defense capacity [30]

**Table 2:** The protective effect of melatonin on mitochondria.

Regulatory Dimension	Core Mechanisms	Key Molecules/ Signaling Pathways	Research Models
Maintenance of ΔΨm	Direct scavenging of ROS/RNS to reduce mitochondrial membrane oxidative damage.	ROS/RNS, mPTP, PGAM5, SIRT1, SIRT3, PGC-1α, PI3K/AKT/Nrf2, Bcl-2 Family, mito-STAT3, GRIM-19, SOD2, cGAS-STING, FGF-21	*In vitro*: Schwann cells (high glucose-induced) [46]; Hippocampal HT22 cells (OGD/R injury) [53];
	Inhibition of mPTP opening.		*In vivo*: Paraquat-Induced PD Model in Male Wistar Rats via Multiple Intraperitoneal Injections [51]; Isoproterenol Hydrochloride-Induced Acute Heart Failure Model in aged male Wistar rats [50]; Hepatic I/R injury model in 8–10 week-old male C57BL/6 mice [48]; Cd exposure model in seven-week-old CD-1 male mice [47];
	Activation of antioxidant signaling pathways.		
	Regulation of the balance of apoptosis-related proteins.		
Regulation of Mitochondrial Quality Control	Modulation of mitochondrial dynamics (inhibiting excessive fission, promoting fusion).	DRP1, Fis1, Mfn1/2, OPA1, Parkin, PINK1, SIRT1, Clock, LC3, Tom20, Beclin1, p62, Bax, cleaved-caspase 3	*In vitro*: RSC96 cells (TBHP-induced injury) [60]; human ovarian granulosa cells [62];
	Context-dependent regulation of mitophagy (activating or inhibiting Parkin/PINK1-dependent selective autophagy).		*In vivo*: Subacute CdCl_2_-Induced Neurotoxicity Model in Adult Male Sprague Dawley Rats via Intraperitoneal Administration [57]; Chronic melatonin treatment model in ZDF rats with diet-induced obesity and type 2 diabetes [58]; Sciatic nerve crush injury model in male Sprague Dawley rats [60]; Photothrombotic infarction model in 8-week-old male C57BL/6J mice [61]; DHT-induced PCOS model in 21-day-old female C57BL/6 mice [62];
	Repair of mitochondrial cristae integrity.		
Promotion of Mitochondrial Biogenesis and Energy Metabolism Remodeling	Activation of upstream regulatory hubs (AMPK, SIRT1) to regulate the PGC-1α core transcriptional axis.	AMPK, SIRT1, PGC-1α, Tfam, NRF2/RCAN/MEF2, β-hydroxyacyl-CoA dehydrogenase, Citrate synthase, Respiratory chain complexes I/II/III/IV	*In vitro*: Primary cultured neonatal mouse ventricular cardiomyocytes [63]; Mesenchymal stem cells [64]; C2C12 myoblasts [66];
	Enhancement of mitochondrial oxidative phosphorylation efficiency.		
	Activation of respiratory chain complex activity.		*In vivo*: Obesity-related type 2 diabetes model in 5-week-old male and female ZDF rats [65];
	Optimization of fatty acid oxidation and regulation of substrate utilization preference.		

**Table 3:** Application of Melatonin in Neurodegenerative Diseases.

Disease	Mitochondrial Targets	Melatonin Mechanisms	Evidence	Pathways in Key Studies	References
Alzheimer’s Disease (AD)	Dysfunction, Oxidative stress; Respiratory deficits; Morphological abnormalitie;	Antioxidant activity, stabilization, inhibition of Aβ/tau aggregation, modulation of autophagy/proteostasis; Restoration of circadian rhythms/sleep; Activates PI3K/Akt to inhibit GSK-3β; Reduces Aβ production, anti-apoptotic; Binds/disaggregates Aβ fibrils; Regulates tau phosphorylation via miR-504-3p, PP2A activation, SIRT1/SIRT3 deacetylation; Activates SIRT1/Nrf2/HO-1 to reduce Aβ/synaptic dysfunction;	Cell (e.g., SH-SY5Y cells); Animal (e.g., streptozotocin rats; hTau mice; high-fat diet models); Human (meta-analysis);	PI3K/Akt/GSK-3β; SIRT1/Nrf2/HO-1;	[84,85,86,87,88,89,90,91,92,93,94,95,96,97]
Parkinson’s Disease (PD)	Dysfunction; Oxidative stress; Fission (via DRP1); Impaired autophagic flux; Reduced complex I activity;	Restores MT1 expression, inhibits mitochondrial fission (PKA/ERK1/2 on DRP1); Improves complex I/respiratory control; Activates PI3K/AKT/Nrf2 to upregulate HO-1/NQO1, attenuates ROS; Dual autophagy regulation (promotes in stem cells via mTOR downregulation for α-syn clearance, suppresses excessive via HSP70/CDK5 inhibition); Anti-inflammatory (upregulates RORα to modulate STAT, reduces TNF-α/IL-1β/IL-6, promotes M2 microglia);	Cell (e.g., AD-MSCs); Animal (e.g., paraquat/MPP+ models; rotenone models implied); Human (meta-analysis);	Oral supplementation; PI3K/AKT/Nrf2;	[98,99,100,101,102,103,104,105,106]
Huntington’s Disease (HD)	Impaired local synthesis (AA-NAT sequestration); Reduced MT1 expression/localization, mtDNA release; oxidative stress; reduced ΔΨm;	Suppresses mtDNA release, downregulates cGAS/STING/IRF3, reduces IL-6/IL-1β; Restores ΔΨm, protects synapses/neurons; Stabilizes neurotransmitters, preserves neuronal morphology; Inhibits caspase activation, prevents pro-apoptotic factor release, protects MT1 from degradation;	Cell (e.g., AA-NAT knockout); Animal (e.g., transgenic mice); Human (small cross-over clinical trial);	Melatonin-MT1 axis; cGAS/STING/IRF3;	[6,107,108,109,110,111,112,113,114,115,116]
Amyotrophic Lateral Sclerosis (ALS)	Dysfunction; Oxidative stress; Respiratory impairment; Cytochrome c release;	Inhibits Rip2/caspase-1, blocks cytochrome c release/caspase-3 activation, restores MT1 expression;	Animal (e.g., SOD1G93A mice); Human (PRO-ACT database analysis);	IP injection; Rip2/caspase-1;	[117,118,119,120,121,122]
Multiple Sclerosis (MS)	Impaired energy metabolism; Neuro-axonal injury;	Upregulates PDK4; Anti-inflammatory/immunomodulatory; Intervenes tryptophan-kynurenine; suppresses nicotinamide N-methyltransferase to elevate NAD^+^;	Animal (e.g., EAE model); Human (observational);	Aryl hydrocarbon receptor; tryptophan-kynurenine;	[123,124,125,126,127]
Prion Diseases	Dynamics/homeostasis; Fragmentation; ROS production; Reduced ATP/ΔΨm;	Modulates DRP1/OPA1 to suppress fragmentation, improves perinuclear distribution; Reduces ROS, restores ATP/ΔΨm; Activates autophagy-lysosomal to clear damaged mitochondria; Blocks Bax translocation, cytochrome c release, p38MAPK/p53 apoptosis;	Cell (e.g., PrP106-126 models); Animal (e.g., delays neuropathology);	Autophagy-lysosomal (LC3-II);	[8,128,129]

## Data Availability

All data discussed in this review are from previously published studies; no new data were generated.
